# HIV drug therapy duration; a Swedish real world nationwide cohort study on InfCareHIV 2009-2014

**DOI:** 10.1371/journal.pone.0171227

**Published:** 2017-02-16

**Authors:** Amanda Häggblom, Stefan Lindbäck, Magnus Gisslén, Leo Flamholc, Bo Hejdeman, Andreas Palmborg, Amy Leval, Eva Herweijer, Sverrir Valgardsson, Veronica Svedhem

**Affiliations:** 1 Department of Infectious Diseases, County Council of Gävleborg, Gävle, Sweden; 2 Unit of Infectious diseases, Department of Medicine Huddinge, Karolinska Institute, Sweden; 3 Janssen Nordics, Solna, Stockholm, Sweden; 4 Department of Infectious Diseases, Sahlgrenska Academy, University of Gothenburg, Gothenburg, Sweden; 5 Department of Infectious Diseases, Malmö University Hospital, Malmö, Sweden; 6 Department of Infectious Diseases, Venhälsan-Södersjukhuset, Stockholm, Sweden; 7 Medical Epidemiology and Biostatistics, Karolinska Institute, Stockholm, Sweden; 8 Department of Infectious Diseases, Karolinska University Hospital, Huddinge, Sweden; Azienda Ospedaliera Universitaria di Perugia, ITALY

## Abstract

**Background:**

As HIV infection needs a lifelong treatment, studying drug therapy duration and factors influencing treatment durability is crucial. The Swedish database InfCareHIV includes high quality data from more than 99% of all patients diagnosed with HIV infection in Sweden and provides a unique opportunity to examine outcomes in a nationwide real world cohort.

**Methods:**

Adult patients who started a new therapy defined as a new 3^rd^ agent (all antiretrovirals that are not N[t]RTIs) 2009–2014 with more than 100 observations in treatment-naive or treatment-experienced patients were included. Dolutegravir was excluded due to short follow up period. Multivariate Cox proportional hazards models were used to estimate hazard ratios for treatment discontinuation.

**Results:**

In treatment-naïve 2541 patients started 2583 episodes of treatments with a 3^rd^ agent. Efavirenz was most commonly used (n = 1096) followed by darunavir (n = 504), atazanavir (n = 386), lopinavir (n = 292), rilpivirine (n = 156) and raltegravir (n = 149). In comparison with efavirenz, patients on rilpivirine were least likely to discontinue treatment (adjusted HR 0.33; 95% CI 0.20–0.54, p<0.001), while patients on lopinavir were most likely to discontinue treatment (adjusted HR 2.80; 95% CI 2.30–3.40, p<0.001). Also raltegravir was associated with early treatment discontinuation (adjusted HR 1.47; 95% CI 1.12–1.92, p = 0.005). The adjusted HR for atazanavir and darunavir were not significantly different from efavirenz. In treatment-experienced 2991 patients started 4552 episodes of treatments with a 3^rd^ agent. Darunavir was most commonly used (n = 1285), followed by atazanavir (n = 806), efavirenz (n = 694), raltegravir (n = 622), rilpivirine (n = 592), lopinavir (n = 291) and etravirine (n = 262). Compared to darunavir all other drugs except for rilpivirine (HR 0.66; 95% CI 0.52–0.83, p<0.001) had higher risk for discontinuation in the multivariate adjusted analyses; atazanavir (HR 1.71; 95% CI 1.48–1.97, p<0.001), efavirenz (HR 1.86; 95% CI 1.59–2.17, p<0.001), raltegravir (HR 1.35; 95% CI 1.15–1.58, p<0.001), lopinavir (HR 3.58; 95% CI 3.02–4.25, p<0.001) and etravirine (HR 1.61; 95% CI 1.31–1.98, p<0.001).Besides the 3^rd^ agent chosen also certain baseline characteristics of patients were independently associated with differences in treatment duration. In naive patients, presence of an AIDS-defining diagnosis and the use of other backbone than TDF/FTC or ABC/3TC increased the risk for early treatment discontinuation. In treatment-experienced patients, detectable plasma viral load at the time of switch or being highly treatment experienced increased the risk for early treatment discontinuation.

**Conclusions:**

Treatment durability is dependent on several factors among others patient characteristics and ART guidelines. The choice of 3^rd^ agent has a strong impact and significant differences between different drugs on treatment duration exist.

## Introduction

Modern HIV treatment has transformed HIV from a fatal disease to a chronic condition. Since cure is not yet possible, combination anti-retroviral treatment (cART) must be lifelong. Despite the success of therapeutic developments in the past decades [1], there are still treatment challenges to overcome; among others transmitted drug resistance, adherence, drug to drug interactions and toxicity [2–6]. Performance and characteristics of antiretroviral (ARV) HIV drug efficacy is well described from randomized clinical trials. However, these trials include a highly selected patient population excluding individuals with anticipated non-adherence e.g. due to drug abuse or psychiatric diseases or patients with interfering concomitant diseases, thereby making the trial populations less representative than the real world patients [7].

To maximize long term treatment outcomes we need to identify the most durable treatment regimens and also investigate other factors independently associated with treatment duration. The Swedish database InfCareHIV includes high quality data from more than 99% of all patients diagnosed with HIV infection in Sweden and provides a unique opportunity to examine outcomes in a nationwide real world cohort [8].

All ARVs approved by the European Medicines Agency (EMA) are available and free of charge for HIV-infected individuals in Sweden. The Swedish HIV treatment guidelines are regularly updated, with the most recent updates being from 2009 [9], 2010 [10], 2011 [11], 2014 [12] and 2016 [13]. Start of first treatment in treatment-naïve patients has been recommended in all patients with a CD4 cell count <350/μL from 2009, for all patients with CD4 cell count 350-500/ μL from 2011 and in all patients, irrespective of CD4 cell counts, from 2014. The recommended backbone nucleoside/nucleotide reverse transcriptase inhibitor (N[t]RTI) treatment in first line has been tenofovir disoproxil fumarate /emtricitabine (TDF/FTC) or abacavir/lamivudine (ABC/3TC), the latter as first alternative in combination with boosted protease inhibitors. Efavirenz (EFV) has been recommended as initial treatment already prior to 2009 and is also recommended in all following guidelines. Rilpivirine (RPV), launched in Sweden 2012, was included as an alternative for patients with HIV-RNA <100 000 copies/mL from 2014. Among protease inhibitors (PIs), boosted atazanavir (ATV) and boosted darunavir (DRV) have been recommended from 2009 while lopinavir (LPV) was excluded as a first line recommendation from 2011 and onwards. Raltegravir (RAL) and dolutegravir (DTG) were included in recommended first line treatments from 2014. No specific recommendations are made in the guidelines regarding the choice of specific drugs in treatment experienced patients, in these the choice of treatment regime is individualized taking into account different variables like reason for switch, prior treatment history, drug resistance and comorbidities.

The aim of the present study was to investigate treatment duration for 3^rd^ agents and factors that might influence duration, in the nationwide HIV cohort in Sweden. Third agents constitute all ARVs that are not N[t]RTIs and that are added to a backbone regimen (BR) of usually two N[t]RTIs. Treatment duration is the time from starting a new 3^rd^ agent to the discontinuation of the same 3^rd^ agent. Treatment duration was chosen as the primary effect outcome as this is a key proxy measure, reflecting the effectiveness, tolerability and convenience of a drug. This study includes all treatment-naïve and treatment-experienced patients from the national InfCareHIV cohort who started a treatment combination with any of the most commonly used 3^rd^ agents from the non-nucleoside reverse transcriptase inhibitor class (NNRTI), the protease inhibitor class (PI) and the integrase inhibitor class (INI) between 2009 to 2014.

## Methods

### Cohort

InfCareHIV has been set-up as a decision support tool in daily clinical care and is also used as a consultation tool for HIV treating physicians, for research purposes and serves also as the National Quality Registry InfCareHIV in Sweden. InfCareHIV was first implemented at Karolinska University Hospital in Stockholm and at Sahlgrenska University Hospital in Gothenburg in 2003 and as of 2009 InfCareHIV was rolled out in all 30 HIV-clinics throughout the country. Patient data, biomarkers, laboratory test results, co-infections and HIV treatments are entered into InfCareHIV, allowing for national follow-up of the care of the Swedish HIV cohort with an estimated coverage of >99% of all patients diagnosed with HIV infection. All HIV patients are actively seen 2–4 times every year and data entry into InfCareHIV is done in conjunction with each clinical visit. For this study, anonymized data was extracted from the existing national registry InfCareHIV.

Naïve or treatment-experienced adult patients who started a new therapy defined as a new 3^rd^ agent between January 1^st^ 2009 and December 31^st^ 2014 were included in the study and followed until the 3^rd^ agent discontinuation, loss to follow up/death or end of study period. Third agents constitute all ARV’s that are not N[t]RTIs and that are added to a backbone regimen of usually two N[t]RTIs.

The integrase inhibitor dolutegravir, launched in Sweden in February 2014, was not included as follow-up time was too short. Patients with HIV-2 infection or under the age 18 were excluded from the analyses.

### Exposure status

Included in the treatment-naive analysis were patients starting their first treatment regimen ever during the study period 2009–2014.

Included in the treatment-experienced analysis were patients with a prior ARV treatment and starting a new treatment including a 3^rd^ agent during 2009–2014. Patients with a prior treatment regimen only including N[t]RTIs and then starting their first regimen including a 3^rd^ agent during the study period were denoted first line treatment experienced patients and patients with one prior 3^rd^ agent containing regimen starting the second 3^rd^ agent during the study period were denoted 2^nd^ line treatment experienced patients. Patients switching 3^rd^ agents during the study period could contribute to several observation periods.

If a 3^rd^ agent was added without withdrawing the previous 3^rd^ agent the patient was still considered on original 3^rd^ agent and a new follow up period was started for the add on 3^rd^ agent. Switch from triple therapy (N[t]RTIs + 3^rd^ agent) to a dual or mono therapy by reducing the N[t]RTIs was still considered the same line of therapy as long as the 3^rd^ agent remained the same. A switch from ritonavir-boosted ATV to non-boosted ATV was considered the same line of therapy. All drugs were dosed according to label; lopinavir and raltegravir twice daily, darunavir once daily in treatment naive patients and once or twice daily in treatment-experienced patients. Treatment interruptions of 30 days or less and then continuing with the same 3^rd^ agent were handled as one treatment sequence in the treatment retention analysis. Treatment interruptions of more than 30 days were considered as a treatment discontinuation.

### Analysis

Descriptive statistics were used to calculate number of patients on treatment. Multivariate Cox proportional hazards models were used to estimate hazard ratios for treatment discontinuation. The most commonly prescribed 3^rd^ agent was used as a reference. For treatment-naïve patients, it was efavirenz and for treatment-experienced patients, it was darunavir. Variables collected in the InfCareHIV cohort and with a known or theoretical relationship to either the exposure or outcome, or both, were included in the analysis to assess for potential confounding biases: age, gender, mode of transmission, region of birth, CDC class, hepatitis status, year of treatment start, baseline CD4, baseline viral load, backbone treatment used, and for treatment experienced patients also line of therapy and years since start of first ARV treatment. Early treatment discontinuation refers to covariates or 3^rd^ agents with a significantly higher risk (hazard ratio) for treatment discontinuation in the multivariate Cox regression model compared to the reference. For region of birth we used a modified UNAIDS regional classification dividing the regions in 4 geographical areas; 1. Sweden, 2. Western Europe, USA, Canada, Israel, Middle East, North Africa, 3. Africa East, South, West, Central, 4. Eastern Europe, Asia, Pacific, Caribbean, and Latin America. If a CD4 count or viral load was missing at treatment start laboratory results from the first week of therapy or from the preceding 6 months were used as baseline values.

Analyses are stratified by treatment-naive and treatment-experienced individuals. Only treatments with more than 100 observations of initiation or change of a 3^rd^ agent in treatment-naive or treatment-experienced patients were included in the analyses. Adjusted and unadjusted hazard ratios (HR) with corresponding 95% confidence intervals (CI) are shown. Treatment discontinuation rates were calculated (1-Kaplan Meier *100%) overall and per therapy for both naïve and experienced patient populations at 90 days, one year, two years and three years after start of treatment. Data were analyzed using SAS version 9.2.

### Ethical considerations

The InfCareHIV registry has ethical approval for studies with retrospective analyses on de-identified patient data (Regional Ethical Review Board, University of Gothenburg Dnr 532–11).

## Results

After exclusions a total of 4724 patients and 7142 observations were included in the analyses. Among treatment-naïve, 2537 patients corresponding to 2583 observations and among treatment-experienced, 2991 patients corresponding to 4552 observations were included. 46 treatment-naive patients started a regimen including two 3^rd^ agents. The 3^rd^ agents initiated by more than 100 treatment naive patients during the study period and thereby included in the analysis were efavirenz, rilpivirine, lopinavir, atazanavir, darunavir and raltegravir. The same antiretroviral drugs were included in the analyses of treatment-experienced patients with the addition of etravirine. The mean 3^rd^ agent treatment observation time for treatment naive and treatment experienced patients were 28.1 and 28.2 months, respectively.

### Baseline characteristics

#### Treatment-naive patients

In treatment-naïve 2537 patients initiating cART with a 3^rd^ agent were included in the study during the six year study period 2009–2014. They were predominately male (63%) and 81% were less than 50 years old at treatment start. Transmission route was heterosexual contact in 51%, men who have sex with men (MSM) in 31%, intravenous drug use (IVDU) in 6% and other/unknown in 8%. Data on transmission route was missing in 3% of cases. Thirty-six percent of the patients had African origin, 34% were from Sweden, 16% Asian/Eastern Euroupe and 14% were from Western Europe/North America. In eight percent of the observations the patient had a history of AIDS diagnosis at start of their first ART. Efavirenz was the most commonly used 3^rd^ agent (n = 1096) followed by darunavir (n = 504), atazanavir (n = 386), lopinavir (n = 292), rilpivirine (n = 156) and raltegravir (n = 149). There were differences in the use of the different ARVs in relation to patient characteristics. This was most clearly seen with rilpivirine that was rarely used in patients with high baseline viral load or low CD4 cell counts; only 3% of the rilpivirine patients had a baseline viral load >100 000 HIV-RNA copies/mL, 3% had a CD4 cell count <200/μL and none of the patients receiving rilpivirine as treatment-naïve had a history of AIDS diagnosis at treatment start. A higher proportion of the patients starting darunavir or lopinavir had a CD4 cell count below 200/μL, and together with raltegravir a higher proportion also had an AIDS diagnosis at start of treatment. Patients starting darunavir or raltegravir more often had a viral load >100 000 copies/mL. Lopinavir and atazanavir were significantly more often used in women. A full description of baseline characteristics for treatment-naive patients can be found in Table 1.

**Table 1:** Baseline characteristics for treatment-naive patients.

Covariate	Value	Efavirenz (n = 1096)	Rilpivirine (n = 156)	Lopinavir (n = 292)	Atazanavir (n = 386)	Darunavir (n = 504)	Raltegravir (n = 149)	Total (n = 2583)
Age	Age <50	878 (80.1%)	133 (85.3%)	258 (88.4%)	317 (82.1%)	401 (79.6%)	114 (76.5%)	2101 (81.3%)
	Age ≥50	218 (19.9%)	23 (14.7%)	34 (11.6%)	69 (17.9%)	102 (20.2%)	35 (23.5%)	481 (18.6%)
	Missing					1 (0.2%)		1 (0.0%)
NRTI Backbone	No Backbone	4 (0.4%)	1 (0.6%)	3 (1.0%)	3 (0.8%)	36 (7.1%)	27 (18.1%)	74 (2.9%)
	ABC/3TC	183 (16.7%)	4 (2.6%)	69 (23.6%)	162 (42.0%)	166 (32.9%)	14 (9.4%)	598 (23.2%)
	TDF/FTC	863 (78.7%)	151 (96.8%)	102 (34.9%)	205 (53.1%)	298 (59.1%)	102 (68.5%)	1721 (66.6%)
	Other	46 (4.2%)		118 (40.4%)	16 (4.1%)	4 (0.8%)	6 (4.0%)	190 (7.4%)
CD4 cell count at baseline	≤200	316 (28.8%)	5 (3.2%)	104 (35.6%)	110 (28.5%)	175 (34.7%)	43 (28.9%)	753 (29.2%)
	201–350	394 (35.9%)	39 (25.0%)	90 (30.8%)	152 (39.4%)	146 (29.0%)	28 (18.8%)	849 (32.9%)
	351–500	207 (18.9%)	58 (37.2%)	47 (16.1%)	70 (18.1%)	106 (21.0%)	45 (30.2%)	533 (20.6%)
	>500	88 (8.0%)	47 (30.1%)	31 (10.6%)	37 (9.6%)	50 (9.9%)	24 (16.1%)	277 (10.7%)
	Missing	91 (8.3%)	7 (4.5%)	20 (6.8%)	17 (4.4%)	27 (5.4%)	9 (6.0%)	171 (6.6%)
CDC Class	C/AIDS	75 (6.8%)		42 (14.4%)	27 (7.0%)	53 (10.5%)	21 (14.1%)	218 (8.4%)
	Non-C (i.e. A or B)	1021 (93.2%)	156 (100.0%)	250 (85.6%)	359 (93.0%)	451 (89.5%)	128 (85.9%)	2365 (91.6%)
Region of birth	Sweden	357 (32.6%)	62 (39.7%)	57 (19.5%)	125 (32.4%)	201 (39.9%)	64 (43.0%)	866 (33.5%)
	Western Europe, USA, Israel, Canada, North Africa, Middle East	152 (13.9%)	38 (24.4%)	18 (6.2%)	46 (11.9%)	71 (14.1%)	33 (22.1%)	358 (13.9%)
	Africa (East, South, West and Central)	405 (37.0%)	28 (17.9%)	157 (53.8%)	147 (38.1%)	147 (29.2%)	36 (24.2%)	920 (35.6%)
	Eastern Europe, Asia, Pacific, Caribbean, Latin America	168 (15.3%)	25 (16.0%)	57 (19.5%)	63 (16.3%)	74 (14.7%)	15 (10.1%)	402 (15.6%)
	Missing	14 (1.3%)	3 (1.9%)	3 (1.0%)	5 (1.3%)	11 (2.2%)	1 (0.7%)	37 (1.4%)
Gender	Male	779 (71.1%)	120 (76.9%)	90 (30.8%)	202 (52.3%)	334 (66.3%)	105 (70.5%)	1630 (63.1%)
	Female	317 (28.9%)	36 (23.1%)	202 (69.2%)	184 (47.7%)	170 (33.7%)	44 (29.5%)	953 (36.9%)
								
Hepatitis status	Negative	1066 (97.3%)	151 (96.8%)	280 (95.9%)	360 (93.3%)	477 (94.6%)	145 (97.3%)	2479 (96.0%)
	Hep B seropositive	14 (1.3%)	2 (1.3%)	3 (1.0%)	4 (1.0%)	6 (1.2%)	1 (0.7%)	30 (1.2%)
	Hep C seropositive	16 (1.5%)	3 (1.9%)	8 (2.7%)	22 (5.7%)	19 (3.8%)	3 (2.0%)	71 (2.7%)
	Hep B+C seropositive			1 (0.3%)		2 (0.4%)		3 (0.1%)
Transmission Route	Heterosexual	572 (52.2%)	52 (33.3%)	193 (66.1%)	219 (56.7%)	229 (45.4%)	52 (34.9%)	1317 (51.0%)
	MSM	353 (32.2%)	90 (57.7%)	27 (9.2%)	83 (21.5%)	176 (34.9%)	81 (54.4%)	810 (31.4%)
	IVDU	39 (3.6%)	5 (3.2%)	23 (7.9%)	35 (9.1%)	44 (8.7%)	5 (3.4%)	151 (5.8%)
	Other/unknown	100 (9.1%)	4 (2.6%)	42 (14.4%)	38 (9.8%)	26 (5.2%)	9 (6.0%)	219 (8.5%)
	Missing	32 (2.9%)	5 (3.2%)	7 (2.4%)	11 (2.8%)	29 (5.8%)	2 (1.3%)	86 (3.3%)
Treatment Start Year	2009–2010	449 (41.0%)	4 (2.6%)	202 (69.2%)	197 (51.0%)	53 (10.5%)	28 (18.8%)	933 (36.1%)
	2011–2012	384 (35.0%)	38 (24.4%)	72 (24.7%)	116 (30.1%)	228 (45.2%)	52 (34.9%)	890 (34.5%)
	2013–2014	263 (24.0%)	114 (73.1%)	18 (6.2%)	73 (18.9%)	223 (44.2%)	69 (46.3%)	760 (29.4%)
Baseline Viral Load	<50	19 (1.7%)	4 (2.6%)	13 (4.5%)	7 (1.8%)	9 (1.8%)	5 (3.4%)	57 (2.2%)
	51–1,000	48 (4.4%)	14 (9.0%)	21 (7.2%)	18 (4.7%)	22 (4.4%)	7 (4.7%)	130 (5.0%)
	1,001–10,000	143 (13.0%)	62 (39.7%)	54 (18.5%)	62 (16.1%)	70 (13.9%)	16 (10.7%)	407 (15.8%)
	10,001–100,000	407 (37.1%)	65 (41.7%)	105 (36.0%)	150 (38.9%)	191 (37.9%)	60 (40.3%)	978 (37.9%)
	>100,000	359 (32.8%)	4 (2.6%)	73 (25.0%)	120 (31.1%)	186 (36.9%)	51 (34.2%)	793 (30.7%)
	Missing	120 (10.9%)	7 (4.5%)	26 (8.9%)	29 (7.5%)	26 (5.2%)	10 (6.7%)	218 (8.4%)
Age (median, p25-p75)		39 (32–47)	37 (31–46)	35 (29–41)	37 (31–45)	39 (32–48)	40 (33–48)	38 (31–47)
Year Start 1st Line Treatment (median, p25-p75)		2011 (2010–2012)	2013 (2012–2013)	2010 (2009–2011)	2010 (2010–2012)	2012 (2011–2013)	2012 (2011–2014)	2011 (2010–2013)

#### Treatment-experienced patients

During the 6 year observation period 2991 treatment-experienced patients started 4552 episodes of treatments with a 3^rd^ agent. At treatment start 58% were male. Transmission route and country of origin were similar to treatment-naive patients. 18% had a history of AIDS at start of treatment and 13% had a CD4 cell count below 200/μL. 57% of the treatments started in a patient with a viral load <50 HIV-RNA copies/mL. In 37% of the observations the patients started a 2nd line treatment, in 23% a 3^rd^ line and in 39% the patient started treatment line 4 or higher. Darunavir was the mostly used ART with 1285 observed treatments followed by 806 observed treatments with atazanavir. A full description of baseline characteristics for treatment-experienced patient can be found in Table 2.

**Table 2:** Baseline characteristics for treatment-experienced patients.

Covariate	Value	Efavirenz (n = 694)	Etravirine (n = 262)	Rilpivirine (n = 592)	Lopinavir (n = 291)	Atazanavir (n = 806)	Darunavir (n = 1285)	Raltegravir (n = 622)	Total (n = 4552)
Age	Age <50	536 (77.2%)	164 (62.6%)	404 (68.2%)	212 (72.9%)	602 (74.7%)	868 (67.5%)	358 (57.6%)	3144 (69.1%)
	Age ≥50	158 (22.8%)	98 (37.4%)	188 (31.8%)	77 (26.5%)	204 (25.3%)	416 (32.4%)	262 (42.1%)	1403 (30.8%)
	Missing				2 (0.7%)		1 (0.1%)	2 (0.3%)	5 (0.1%)
NRTI Backbone	No Backbone	4 (0.6%)	77 (29.4%)	8 (1.4%)	18 (6.2%)	8 (1.0%)	137 (10.7%)	104 (16.7%)	356 (7.8%)
	ABC/3TC	128 (18.4%)	38 (14.5%)	115 (19.4%)	95 (32.6%)	379 (47.0%)	447 (34.8%)	174 (28.0%)	1376 (30.2%)
	TDF/FTC	530 (76.4%)	106 (40.5%)	461 (77.9%)	107 (36.8%)	385 (47.8%)	601 (46.8%)	273 (43.9%)	2463 (54.1%)
	Other	32 (4.6%)	41 (15.6%)	8 (1.4%)	71 (24.4%)	34 (4.2%)	100 (7.8%)	71 (11.4%)	357 (7.8%)
CD4 cell count at baseline	≤200	73 (10.5%)	34 (13.0%)	25 (4.2%)	54 (18.6%)	107 (13.3%)	220 (17.1%)	93 (15.0%)	606 (13.3%)
	201–350	153 (22.0%)	48 (18.3%)	70 (11.8%)	87 (29.9%)	216 (26.8%)	268 (20.9%)	102 (16.4%)	944 (20.7%)
	351–500	165 (23.8%)	61 (23.3%)	123 (20.8%)	56 (19.2%)	188 (23.3%)	283 (22.0%)	116 (18.6%)	992 (21.8%)
	>500	247 (35.6%)	94 (35.9%)	290 (49.0%)	68 (23.4%)	236 (29.3%)	421 (32.8%)	250 (40.2%)	1606 (35.3%)
	Missing	56 (8.1%)	25 (9.5%)	84 (14.2%)	26 (8.9%)	59 (7.3%)	93 (7.2%)	61 (9.8%)	404 (8.9%)
CDC Class	C/AIDS	105 (15.1%)	71 (27.1%)	79 (13.3%)	60 (20.6%)	124 (15.4%)	238 (18.5%)	146 (23.5%)	823 (18.1%)
	Non-C (i.e. A or B)	589 (84.9%)	191 (72.9%)	513 (86.7%)	231 (79.4%)	682 (84.6%)	1047 (81.5%)	476 (76.5%)	3729 (81.9%)
Region of birth	Sweden	222 (32.0%)	140 (53.4%)	260 (43.9%)	102 (35.1%)	293 (36.4%)	496 (38.6%)	303 (48.7%)	1816 (39.9%)
	Western Europe, USA, Israel, Canada, North Africa, Middle East	78 (11.2%)	43 (16.4%)	104 (17.6%)	20 (6.9%)	80 (9.9%)	161 (12.5%)	90 (14.5%)	576 (12.7%)
	Africa (East, South, West and Central)	284 (40.9%)	58 (22.1%)	151 (25.5%)	126 (43.3%)	323 (40.1%)	485 (37.7%)	171 (27.5%)	1598 (35.1%)
	Eastern Europe, Asia, Pacific, Caribbean, Latin America	102 (14.7%)	21 (8.0%)	66 (11.1%)	41 (14.1%)	106 (13.2%)	133 (10.4%)	52 (8.4%)	521 (11.4%)
	Missing	8 (1.2%)		11 (1.9%)	2 (0.7%)	4 (0.5%)	10 (0.8%)	6 (1.0%)	41 (0.9%)
Gender	Male	365 (52.6%)	195 (74.4%)	398 (67.2%)	129 (44.3%)	404 (50.1%)	753 (58.6%)	407 (65.4%)	2651 (58.2%)
	Female	329 (47.4%)	67 (25.6%)	194 (32.8%)	162 (55.7%)	402 (49.9%)	532 (41.4%)	215 (34.6%)	1901 (41.8%)
									
Hepatitis status	Negative	651 (93.8%)	251 (95.8%)	554 (93.6%)	271 (93.1%)	733 (90.9%)	1197 (93.2%)	584 (93.9%)	4241 (93.2%)
	Hep B seropositive	18 (2.6%)	5 (1.9%)	13 (2.2%)	1 (0.3%)	10 (1.2%)	27 (2.1%)	12 (1.9%)	86 (1.9%)
	Hep C seropositive	23 (3.3%)	6 (2.3%)	23 (3.9%)	18 (6.2%)	61 (7.6%)	56 (4.4%)	26 (4.2%)	213 (4.7%)
	Hep B+C seropositive	2 (0.3%)		2 (0.3%)	1 (0.3%)	2 (0.2%)	5 (0.4%)		12 (0.3%)
Transmission Route	Heterosexual	416 (59.9%)	96 (36.6%)	263 (44.4%)	181 (62.2%)	464 (57.6%)	669 (52.1%)	268 (43.1%)	2357 (51.8%)
	MSM	186 (26.8%)	142 (54.2%)	272 (45.9%)	44 (15.1%)	168 (20.8%)	366 (28.5%)	276 (44.4%)	1454 (31.9%)
	IVDU	28 (4.0%)	10 (3.8%)	17 (2.9%)	38 (13.1%)	106 (13.2%)	129 (10.0%)	30 (4.8%)	358 (7.9%)
	Other/unknown	60 (8.6%)	13 (5.0%)	36 (6.1%)	23 (7.9%)	62 (7.7%)	114 (8.9%)	45 (7.2%)	353 (7.8%)
	Missing	4 (0.6%)	1 (0.4%)	4 (0.7%)	5 (1.7%)	6 (0.7%)	7 (0.5%)	3 (0.5%)	30 (0.7%)
Treatment Start Year	2009–2010	377 (54.3%)	121 (46.2%)		194 (66.7%)	424 (52.6%)	327 (25.4%)	211 (33.9%)	1654 (36.3%)
	2011–2012	218 (31.4%)	80 (30.5%)	202 (34.1%)	74 (25.4%)	266 (33.0%)	545 (42.4%)	232 (37.3%)	1617 (35.5%)
	2013–2014	99 (14.3%)	61 (23.3%)	390 (65.9%)	23 (7.9%)	116 (14.4%)	413 (32.1%)	179 (28.8%)	1281 (28.1%)
Line of Therapy	1*	30 (4.3%)	1 (0.4%)	5 (0.8%)	5 (1.7%)	13 (1.6%)	8 (0.6%)	4 (0.6%)	66 (1.4%)
	2	352 (50.7%)	31 (11.8%)	261 (44.1%)	119 (40.9%)	336 (41.7%)	391 (30.4%)	157 (25.2%)	1647 (36.2%)
	3	164 (23.6%)	43 (16.4%)	130 (22.0%)	75 (25.8%)	204 (25.3%)	311 (24.2%)	130 (20.9%)	1057 (23.2%)
	4+	148 (21.3%)	187 (71.4%)	196 (33.1%)	92 (31.6%)	253 (31.4%)	575 (44.7%)	331 (53.2%)	1782 (39.1%)
Baseline Viral Load	<50	433 (62.4%)	136 (51.9%)	515 (87.0%)	98 (33.7%)	424 (52.6%)	649 (50.5%)	358 (57.6%)	2613 (57.4%)
	51–1,000	65 (9.4%)	59 (22.5%)	31 (5.2%)	49 (16.8%)	98 (12.2%)	236 (18.4%)	120 (19.3%)	658 (14.5%)
	1,001–10,000	55 (7.9%)	18 (6.9%)	18 (3.0%)	36 (12.4%)	68 (8.4%)	102 (7.9%)	41 (6.6%)	338 (7.4%)
	10,001–100,000	76 (11.0%)	29 (11.1%)	13 (2.2%)	53 (18.2%)	116 (14.4%)	165 (12.8%)	60 (9.6%)	512 (11.2%)
	>100,000	34 (4.9%)	16 (6.1%)	3 (0.5%)	37 (12.7%)	65 (8.1%)	110 (8.6%)	26 (4.2%)	291 (6.4%)
	Missing	31 (4.5%)	4 (1.5%)	12 (2.0%)	18 (6.2%)	35 (4.3%)	23 (1.8%)	17 (2.7%)	140 (3.1%)
Years Since Start of first ART	0–2 years	278 (40.1%)	56 (21.4%)	190 (32.1%)	108 (37.1%)	289 (35.9%)	327 (25.4%)	165 (26.5%)	1413 (31.0%)
	3–5 years	106 (15.3%)	23 (8.8%)	101 (17.1%)	48 (16.5%)	122 (15.1%)	170 (13.2%)	53 (8.5%)	623 (13.7%)
	5+ years	310 (44.7%)	183 (69.8%)	301 (50.8%)	135 (46.4%)	395 (49.0%)	788 (61.3%)	404 (65.0%)	2516 (55.3%)
Age (median, p25-p75)		41 (34–49)	47 (41–56)	44 (37–53)	41 (34–50)	42 (35–50)	44 (37–52)	47 (40–56)	44 (36–52)
Year Start of first ART (median, p25-p75)		2006 (2001–2009)	2000 (1997–2007)	2008 (2002–2011)	2006 (2001–2008)	2006 (2002–2009)	2005 (1998–2009)	2003 (1998–2009)	2005 (1999–2009)

*Includes patients with a prior treatment episode including only N[t]RTIs and starting their first treatment including a 3^rd^ agent during the study period 2009–2014

#### Overall treatment discontinuation rates

Ten percent of treatment naive patients had discontinued the 3^rd^ agent within 90 days from start of treatment. After one year 24% had discontinued, after two years 33% and after three years 42%. Among treatment-experienced patients overall discontinuation rates were very similar; 11% after 90 days, 24% after one year, 34% after two years and 41% after three years. Discontinuation rates varied widely between drugs with the highest discontinuation rates seen with lopinavir; approximately half of the patients, both in treatment-naïve and treatment-experienced patients had discontinued lopinavir within one year from treatment start. The lowest discontinuation rate was seen in patients treated with rilpivirine where only 7% and 12% of treatment-naive and treatment-experienced patients respectively discontinued within one year. Discontinuation rates for all drugs can be found in Table 3.

**Table 3:** Discontinuation rates (1-KM*100%) for treatment-naive and treatment-experienced patient populations.

Patient Population	Treatment	90 days	1 year	2 years	3 years
Naive	Efavirenz	0.10 (0.09;0.12)	0.21 (0.19;0.24)	0.29 (0.26;0.32)	0.35 (0.32;0.38)
	Rilpivirine	0.04 (0.02;0.08)	0.07 (0.04;0.13)	0.11 (0.07;0.18)	0.17 (0.10;0.28)
	Lopinavir	0.16 (0.13;0.21)	0.52 (0.46;0.58)	0.69 (0.64;0.74)	0.79 (0.74;0.83)
	Atazanavir	0.10 (0.07;0.14)	0.22 (0.18;0.26)	0.29 (0.25;0.34)	0.36 (0.31;0.41)
	Darunavir	0.07 (0.05;0.09)	0.18 (0.15;0.22)	0.28 (0.24;0.33)	0.41 (0.36;0.47)
	Raltegravir	0.13 (0.09;0.20)	0.33 (0.26;0.42)	0.43 (0.35;0.53)	0.59 (0.50;0.69)
	Total	0.10 (0.09;0.11)	0.24 (0.22;0.26)	0.33 (0.32;0.35)	0.42 (0.40;0.44)
Experienced	Efavirenz	0.16 (0.13;0.19)	0.30 (0.27;0.34)	0.38 (0.35;0.42)	0.44 (0.40;0.48)
	Etravirine	0.17 (0.13;0.22)	0.29 (0.24;0.35)	0.41 (0.35;0.47)	0.47 (0.40;0.53)
	Rilpivirine	0.06 (0.04;0.08)	0.12 (0.09;0.15)	0.16 (0.13;0.20)	0.22 (0.18;0.28)
	Lopinavir	0.20 (0.16;0.25)	0.50 (0.44;0.56)	0.68 (0.63;0.74)	0.76 (0.71;0.81)
	Atazanavir	0.10 (0.08;0.12)	0.25 (0.22;0.28)	0.38 (0.34;0.41)	0.45 (0.42;0.49)
	Darunavir	0.07 (0.06;0.09)	0.18 (0.16;0.20)	0.28 (0.25;0.30)	0.33 (0.30;0.36)
	Raltegravir	0.11 (0.08;0.13)	0.26 (0.23;0.30)	0.34 (0.30;0.38)	0.41 (0.37;0.46)
	Total	0.11 (0.10;0.12)	0.24 (0.23;0.25)	0.34 (0.33;0.36)	0.41 (0.39;0.42)

### Analysis of treatment duration

#### Treatment-naïve patients

**Fig 1:**
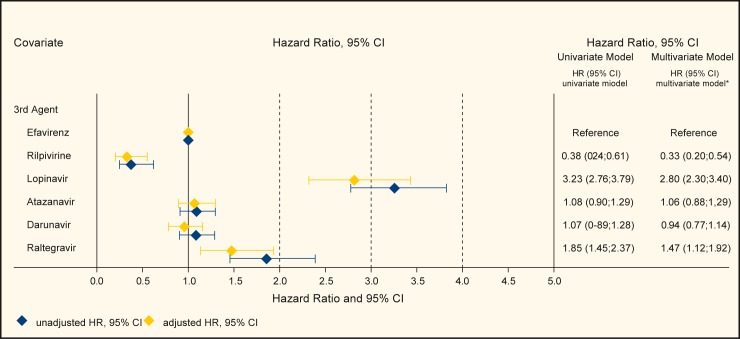
Risk for treatment discontinuation in treatment-naive patients (Treatment effects showing Hazard Ratios from univariate and multivariate Cox regression models).

Among treatment-naïve patients the 3^rd^ agents had different discontinuation rates. In comparison with efavirenz, patients on rilpivirine were least likely to discontinue treatment (adjusted HR 0.33; 95% CI 0.20–0.54, p<0.001), while patients on lopinavir were most likely to discontinue treatment (adjusted HR 2.80; 95% CI 2.30–3.40, p<0.001), see Fig 1A. Also raltegravir was associated with early treatment discontinuation (adjusted HR 1.47; 95% CI 1.12–1.92, p = 0.005). The adjusted HR for atazanavir and darunavir were not significantly different from efavirenz. Hazard ratios for treatment-naive patients can be seen in Fig 1.

Patients with an AIDS diagnosis at treatment start had a significantly higher risk for early treatment discontinuation than non-AIDS patients. Other co-variables independently associated with early treatment discontinuation in the multivariate adjusted model were N[t]RTI backbone other than ABC/3TCor TAF/FTC and treatment start year 2011 or later compared to treatment start 2009–2010.

When transmission route was known to be heterosexual, patients were less likely to discontinue 3^rd^ agent treatment than MSM (adjusted HR 0.67; 95% CI 0.56–0.80, p<0.001). Age, gender, hepatitis status, region of birth, CD4 cell count <200/μL and viral load >100.000 HIV-RNA copies/mL at treatment start were not correlated with treatment discontinuation (Table 4).

**Table 4:** Hazard Ratios from univariate and multivariate Cox regression models for treatment-naive patients.

Covariate	Value	Distinct Patients (n)	Observations (n)	HR (95% CI) univariate model	P-value	HR (95% CI) multivariate model*	P-value
Age	Age <50	2068	2101	Reference		Reference	
	Age ≥50	472	481	0.88 (0.75;1.03)	0.111	0.95 (0.80;1.12)	0.548
	Missing	1	1	10.58 (1.50;74.82)	0.018	23.78 (3.21;175.91)	0.002
N[t]RTI Backbone	No Backbone	50	74	1.57 (1.16;2.13)	0.004	1.24 (0.89;1.73)	0.210
	ABC/3TC	597	598	1.16 (1.00;1.35)	0.045	1.06 (0.91;1.25)	0.439
	TDF/FTC	1708	1721	Reference		Reference	
	Other	186	190	3.01 (2.51;3.61)	<0.001	2.16 (1.72;2.71)	<0.001
CD4 cell count at Baseline	≤200	736	753	0.90 (0.73;1.12)	0.358	0.83 (0.65;1.05)	0.127
	201–350	840	849	0.73 (0.59;0.90)	0.004	0.77 (0.62;0.97)	0.025
	351–500	524	533	0.86 (0.68;1.08)	0.187	0.87 (0.69;1.10)	0.260
	>500	272	277	Reference		Reference	
	Missing	169	171	0.66 (0.49;0.90)	0.008	0.60 (0.39;0.94)	0.024
CDC Class	C/AIDS	207	218	1.56 (1.29;1.88)	<0.001	1.42 (1.15;1.76)	0.001
	Non-C (i.e. A or B)	2334	2365	Reference		Reference	
Region of birth	Sweden	849	866	Reference		Reference	
	Western Europe, USA, Israel, Canada, North Africa, Middle East	353	358	0.98 (0.81;1.19)	0.861	1.00 (0.82;1.21)	0.970
	Africa (East, South, West and Central)	901	920	1.06 (0.92;1.22)	0.436	1.08 (0.90;1.30)	0.409
	Eastern Europe, Asia, Pacific, Caribbean, Latin America	401	402	0.91 (0.75;1.10)	0.316	0.85 (0.69;1.05)	0.141
	Missing	37	37	0.65 (0.35;1.22)	0.182	0.83 (0.43;1.62)	0.587
Gender	Male	1608	1630	Reference		Reference	
	Female	933	953	1.13 (1.00;1.27)	0.060	1.02 (0.86;1.21)	0.809
Hepatitis Status	Negative	2438	2479	Reference		Reference	
	Hep B seropositive	30	30	0.84 (0.46;1.52)	0.565	0.88 (0.49;1.61)	0.688
	Hep C seropositive	70	71	1.16 (0.83;1.61)	0.381	1.21 (0.84;1.73)	0.314
	Hep B+C seropositive	3	3	2.07 (0.52;8.27)	0.305	1.75 (0.43;7.17)	0.434
Transmission Route	Heterosexual	1293	1317	0.86 (0.75;0.99)	0.029	0.67 (0.56;0.80)	<0.001
	MSM	796	810	Reference		Reference	
	IVDU	151	151	0.95 (0.73;1.23)	0.703	0.78 (0.58;1.04)	0.094
	Other/unknown	215	219	1.15 (0.92;1.43)	0.211	0.82 (0.64;1.06)	0.139
	Missing	86	86	0.61 (0.39;0.93)	0.023	0.56 (0.35;0.89)	0.015
Treatment Start Year	2009–2010	926	933	Reference		Reference	
	2011–2012	865	890	0.98 (0.85;1.12)	0.765	1.16 (1.00;1.35)	0.048
	2013–2014	750	760	0.84 (0.70;1.00)	0.049	1.26 (1.03;1.54)	0.023
Baseline Viral Load	<50	54	57	Reference		Reference	
	51–1,000	128	130	0.83 (0.50;1.37)	0.458	1.09 (0.65;1.81)	0.749
	1,001–10,000	405	407	0.86 (0.55;1.34)	0.493	1.15 (0.73;1.82)	0.536
	10,001–100,000	957	978	0.83 (0.54;1.28)	0.399	1.11 (0.72;1.73)	0.639
	>100,000	781	793	1.00 (0.65;1.53)	0.986	1.33 (0.85;2.08)	0.214
	Missing	216	218	0.75 (0.47;1.20)	0.236	1.06 (0.62;1.82)	0.821
3rd Agent	Efavirenz	1096	1096	Reference		Reference	
	Rilpivirine	156	156	0.38 (0.24;0.61)	<0.001	0.33 (0.20;0.54)	<0.001
	Lopinavir	292	292	3.23 (2.76;3.79)	<0.001	2.80 (2.30;3.40)	<0.001
	Atazanavir	386	386	1.08 (0.90;1.29)	0.416	1.06 (0.88;1.29)	0.528
	Darunavir	504	504	1.07 (0.89;1.28)	0.467	0.94 (0.77;1.14)	0.516
	Raltegravir	149	149	1.85 (1.45;2.37)	<0.001	1.47 (1.12;1.92)	0.005

* Adjustments made for treatment, age, gender, region of birth, CD4 count at baseline, viral load at baseline, CDC class, route of infection, year of initiation treatment, hepatitis status, N[t]RTI backbone treatment.

#### Treatment-experienced patients

**Fig 2:**
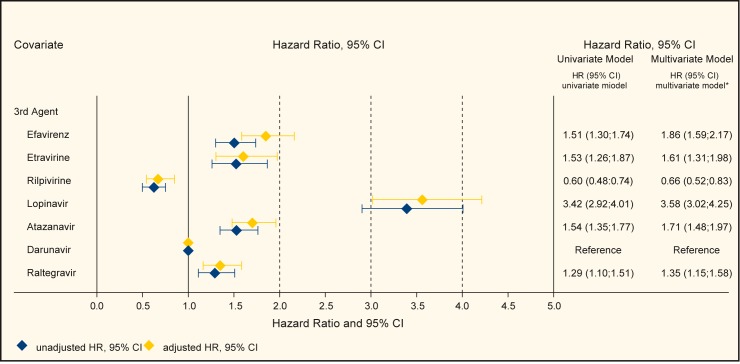
Risk for treatment discontinuation in treatment-experienced patients (Treatment effects showing Hazard Ratios from univariate and multivariate Cox regression models).

Also among treatment-experienced patients, the use of different 3^rd^ agents showed significant different correlations to treatment discontinuation. With darunavir as the reference, patients on rilpivirine had significantly lower discontinuation rates (adjusted HR 0.66; 95% CI 0.52–0.83, p<0.001) and all other drugs had significantly higher risk for discontinuation in the multivariate adjusted analyses; efavirenz (HR 1.86; 95% CI 1.59–2.17, p<0.001), etravirine (HR 1.61; 95% CI 1.31–1.98, p<0.001), lopinavir (HR 3.58; 95% CI 3.02–4.25, p<0.001), atazanavir (HR 1.71; 95% CI 1.48–1.97, p<0.001), and raltegravir (HR 1.35; 95% CI 1.15–1.58, p<0.001) (see Fig 2).

Having a CD4 cell count <200 μL or a viral load >50 HIV-RNA copies/mL at treatment start significantly increased the risk for early treatment discontinuation in the adjusted analyses. Female gender, treatment line 4+ or treatment start 2011 or later also increased the risk. Similar to findings in treatment naive patients, heterosexual transmission route correlated to a lower risk and having a backbone other than ABC/3TCor TDF/FTC correlated to a higher risk for early discontinuation of the 3^rd^ agent. Treatment experienced patients with no backbone had a lower risk for early treatment discontinuation as well as patients with Asian origin. For complete results, see Table 5.

**Table 5:** Hazard Ratios from univariate and multivariate Cox regression models for treatment-experienced patients.

Covariate	Value	Distinct Patients (n)	Observations (n)	HR (95% CI) univariate model	P-value	HR (95% CI) multivariate model*	P-value
Age	Age <50	2081	3144	Reference		Reference	
	Age ≥50	951	1403	0.95 (0.86;1.05)	0.308	0.97 (0.87;1.08)	0.561
	Missing	3	5	0.73 (0.18;2.93)	0.660	0.42 (0.10;1.68)	0.219
N[t]RTI Backbone	No Backbone	211	356	0.84 (0.70;1.01)	0.067	0.81 (0.66;0.99)	0.038
	ABC/3TC	1046	1376	1.06 (0.96;1.17)	0.253	1.09 (0.98;1.21)	0.128
	TDF/FTC	1807	2463	Reference		Reference	
	Other	275	357	1.39 (1.19;1.63)	<0.001	1.21 (1.02;1.43)	0.026
CD4 cell count at baseline	≤200	414	606	1.64 (1.44;1.88)	<0.001	1.27 (1.08;1.49)	0.004
	201–350	732	944	1.29 (1.14;1.46)	<0.001	1.00 (0.87;1.14)	0.978
	351–500	826	992	1.06 (0.93;1.20)	0.393	0.96 (0.84;1.09)	0.495
	>500	1236	1606	Reference		Reference	
	Missing	358	404	1.06 (0.88;1.27)	0.530	1.01 (0.83;1.24)	0.898
CDC Class	C/AIDS	520	823	1.06 (0.95;1.19)	0.285	1.03 (0.91;1.16)	0.630
	Non-C (i.e. A or B)	2482	3729	Reference		Reference	
Region of birth	Sweden	1190	1816	Reference		Reference	
	Western Europe, USA, Israel, Canada, North Africa, Middle East	381	576	1.01 (0.88;1.17)	0.858	1.07 (0.92;1.24)	0.363
	Africa (East, South, West and Central)	1024	1598	1.02 (0.92;1.13)	0.722	0.97 (0.84;1.12)	0.652
	Eastern Europe, Asia, Pacific, Caribbean, Latin America	363	521	0.82 (0.69;0.96)	0.013	0.74 (0.62;0.88)	<0.001
	Missing	33	41	0.58 (0.32;1.05)	0.072	0.73 (0.40;1.35)	0.316
Gender	Male	1779	2651	Reference		Reference	
	Female	1212	1901	1.13 (1.03;1.24)	0.007	1.14 (1.01;1.29)	0.030
Hepatitis Status	Negative	2795	4241	Reference		Reference	
	Hep B seropositive	61	86	0.74 (0.52;1.07)	0.108	0.81 (0.56;1.17)	0.266
	Hep C seropositive	134	213	1.30 (1.07;1.58)	0.009	1.03 (0.82;1.29)	0.788
	Hep B+C seropositive	6	12	1.87 (0.93;3.74)	0.078	1.58 (0.78;3.20)	0.204
Transmission Route	Heterosexual	1550	2357	1.06 (0.95;1.17)	0.286	0.86 (0.74;0.99)	0.039
	MSM	960	1454	Reference		Reference	
	IVDU	220	358	1.60 (1.36;1.88)	<0.001	1.20 (0.99;1.46)	0.066
	Other/unknown	233	353	1.09 (0.91;1.31)	0.357	0.88 (0.72;1.09)	0.242
	Missing	28	30	0.74 (0.35;1.55)	0.424	0.61 (0.28;1.30)	0.199
Treatment Start Year	2009–2010	1309	1654	Reference		Reference	
	2011–2012	1286	1617	0.95 (0.86;1.05)	0.319	1.23 (1.11;1.37)	<0.001
	2013–2014	1084	1281	0.83 (0.72;0.95)	0.006	1.32 (1.14;1.53)	<0.001
Line of Therapy	1†	66	66	0.82 (0.54;1.23)	0.331	0.62 (0.41;0.94)	0.023
	2	1647	1647	Reference		Reference	
	3	1057	1057	0.98 (0.87;1.11)	0.752	1.09 (0.96;1.24)	0.169
	4+	1119	1782	1.09 (0.98;1.21)	0.113	1.37 (1.19;1.56)	<0.001
Baseline Viral Load	<50	2021	2613	Reference		Reference	
	51–1,000	517	658	1.37 (1.20;1.56)	<0.001	1.25 (1.09;1.44)	0.001
	1,001–10,000	267	338	1.83 (1.56;2.14)	<0.001	1.59 (1.34;1.88)	<0.001
	10,001–100,000	408	512	1.49 (1.30;1.72)	<0.001	1.21 (1.04;1.41)	0.014
	>100,000	226	291	1.92 (1.63;2.26)	<0.001	1.46 (1.21;1.75)	<0.001
	Missing	124	140	1.26 (0.98;1.63)	0.073	1.00 (0.76;1.33)	0.973
3rd Agent	Efavirenz	668	694	1.51 (1.30;1.74)	<0.001	1.86 (1.59;2.17)	<0.001
	Etravirine	243	262	1.53 (1.26;1.87)	<0.001	1.61 (1.31;1.98)	<0.001
	Rilpivirine	584	592	0.60 (0.48;0.74)	<0.001	0.66 (0.52;0.83)	<0.001
	Lopinavir	271	291	3.42 (2.92;4.01)	<0.001	3.58 (3.02;4.25)	<0.001
	Atazanavir	720	806	1.54 (1.35;1.77)	<0.001	1.71 (1.48;1.97)	<0.001
	Darunavir	1200	1285	Reference		Reference	
	Raltegravir	594	622	1.29 (1.10;1.51)	0.001	1.35 (1.15;1.58)	<0.001
Years Since Start of first ART	0–2 years	1062	1413	Reference		Reference	
	3–5 years	535	623	0.87 (0.75;1.00)	0.057	0.87 (0.75;1.02)	0.078
	5+ years	1668	2516	0.85 (0.77;0.94)	0.002	0.78 (0.68;0.88)	<0.001

* Adjustments made for treatment, age, gender, ethnicity, CD4 count at treatment baseline, viral load at baseline, CDC class, route of infection, year of initiation treatment, years since start 1st treatment, line of therapy, hepatitis status, and N[t]RTI Backbone treatment.

†Patients with a prior treatment episode with only N[t]RTIs and starting their first treatment including a 3^rd^ agent during the study period 2009–2014

## Discussion

As HIV infection needs lifelong treatment, studying treatment duration and factors influencing treatment durability is crucial. This can be done in randomized clinical trials but, for several reasons, a real life cohort study like ours can give additional benefits.

First, in a randomized clinical trial you study a selected, small, homogeneous patient group in a strictly standardized setting in order to be able to attribute the effect to the specific intervention, for example, the efficacy of a drug. This might lead to results less applicable outside the study setting [7]. In the present study we had the unique opportunity to be able to study the effectiveness of the most used HIV treatments in an entire national real world HIV cohort.

Second, in a randomized clinical trial you exclude the possibility for the physician to use his clinical expertise to individualize the treatment according to patient characteristics. As example, in several clinical studies, efavirenz has been shown to have CNS side effects leading to drug discontinuation in a significant number of patients [14–16]. In the clinical setting the physician can decrease the risk for discontinuations by not using efavirenz in patients with factors known to increase the risk for neuropsychiatric side effects [17–19].

Third, a real world cohort gives the possibility to have a significantly longer observation period than in prospective clinical studies. In this study we followed patients up to 6 years which rarely occurs in a clinical trial.

The overall treatment discontinuation rate in this study was similar for treatment naive and treatment experienced patients; 10% of the patients discontinued the 3^rd^ agent within 3 months and a quarter within one year. However, the differences between drugs were significant with discontinuation rates one year after start of treatment of more than 50% in lopinavir patients compared to approximately 10% in rilpivirine patients. It is also noticeable that different drugs in the same class of drugs can perform very differently. This indicates that comparisons between classes of HIV drugs do not take into account the specific characteristics of the individual ARVs and therefore can be misleading.

We found that the selection of a 3^rd^ agent seemed to have a stronger influence on treatment duration than demographic or clinical factors. Both in treatment-naïve and treatment-experienced patients, rilpivirine had a significantly lower risk for early treatment discontinuation compared to the other drugs studied. There may be several factors contributing to this. In treatment-naïve patients starting on rilpivirine very few had an HIV-RNA >100 000 copies/ml at start of treatment confirming its use according to label, very few had low CD4 cell counts and none of the patients had an AIDS diagnosis. In treatment-experienced patients starting rilpivirine a high proportion, 87% of the patients, had a viral load below 50 copies/ml at baseline indicating that the main reasons for switch were tolerability issues or simplification. Both in treatment-naive and treatment-experienced analyses rilpivirine still had a significantly lower risk of discontinuation when adjusted for these factors but it is still not possible to rule out an unmeasured bias e.g. the prescribing physicians estimation of the patient adherence level. Our interpretation is that the superiority shown for rilpivirine is partly due to channeling bias where rilpivirine was chosen for being easy to treat patients with an anticipated good adherence, but its favorable side effect profile shown in phase 3 studies also was a contributing factor [20,21].

Both in treatment-naïve and in treatment-experienced patients starting lopinavir, there was a significant higher risk for early treatment discontinuation compared to all other drugs studied. A plausible explanation is that early in the study period lopinavir was no longer recommended in the Swedish HIV treatment guidelines because of its less favorable gastro-intestinal side effect profile and its more pronounced negative effect on blood lipids compared to the two other recommended protease inhibitors darunavir and atazanavir [11]. Together with the need for twice daily dosing this may have accelerated the switch rate from lopinavir. Also, a relatively high proportion of patients, 40% of naive and 24% of treatment-experienced who used lopinavir did not use or tenofovir disoproxil fumarate/emtricitabine as N[t]RTI backbone treatment, but other (older) combinations which may have further contributed to the poorer result seen. The high use of other backbone combinations may in part be explained by more women taking lopinavir indicating usage during pregnancy.

Treatment durations for efavirenz, darunavir and atazanavir were similar in treatment-naive patients but raltegravir had a significantly higher risk of early treatment discontinuation. In the STARTMRK study patients on raltegravir had significantly fewer drug related adverse events than patients on efavirenz [22]. In our present study naive patients starting efavirenz showed lower risk for early drug discontinuation compared to raltegravir. One explanation may be that physicians followed the recommendation in Swedish treatment guidelines not to initiate efavirenz treatment in patients with psychiatric problems thereby avoiding some treatment discontinuations due to neuropsychiatric adverse events.

In treatment-experienced patients darunavir, the most commonly used 3^rd^ agent during our study period, showed a significantly lower risk for treatment discontinuation than all other ARVs, except for rilpivirine. In line with clinical trials results our interpretation is that this is a reflection of its favorable side effect profile, compared to other boosted protease inhibitors, together with high efficacy and low risk for resistance development across different patient types [23, 24]. In treatment-experienced patients a high genetic barrier and a low risk for resistance development is of importance if the switch is because of previous treatment failure or sub-optimal adherence [25, 26].

In line with other reports this study confirms that, besides the ARV chosen, certain baseline characteristics of patients are independently associated with differences in treatment duration [27–30]. In naive patients we found that AIDS diagnosis and the use of other backbone than ABC/3TCor TDF/FTC increased the risk for early treatment discontinuation. Heterosexual transmission category decreased the risk in comparison to MSM. In treatment-experienced patients, indicators of viral failure or a highly treatment experienced patient increased the risk for early treatment discontinuation; CD4 cell count <200, use of other backbone than ABC/3TC orTDF/FTC, being on 4^th^ or higher line of treatment or having viral load >50 copies ml at start of treatment. In both treatment-naive and treatment-experienced patients, a start of treatment in 2011 or later was correlated to an earlier treatment discontinuation. One possible explanation is that in this time period we had a higher switch rate from older drugs like lopinavir and newer drugs like rilpivirine, elvitegravir and dolutegravir were introduced.

There are several limitations with our study the most important being that we cannot provide the reasons for drug discontinuations. Both viral failure with development of resistance associated mutations and some drug toxicities may limit future treatment options while this is not the case in treatment modifications to lower pill burden or to have less frequent dosing.

We also did not have any data on the level of adherence. Neither measurements of adherence nor physician`s estimates of adherence were available. Estimated or observed adherence probably has an important impact on the choice of new treatment regimens. Another limitation is that not all kinds of data are included in InfCareHIV. It would have been of interest to see e.g. how socioeconomic factors like education, employment, income, marital status, active drug use influence drug duration time.

Last, the data and the results are mostly applicable to the time period studied and to Sweden or other countries with a similar health care environment. Sweden is a country with a low HIV prevalence, and the care of HIV patients in Sweden is highly specialized; all HIV infected patients are linked to specialized HIV care centers with dedicated multidisciplinary teams of physicians, nurses and social workers, Following the Swedish law of Communicable Disease Act all HIV drugs and HIV health care are freely available for patients but also obliges the patients to keep regular contact with the responsible HIV clinic. All this are contributing to the excellent treatment outcomes in Swedish HIV patients, and we believe Sweden is the first country to achieve the UNAIDS/WHO 90-90-90 targets [31].

In conclusion, we found that selection of the 3^rd^ agent is an important factor to maximize treatment duration. The choice of backbone, ABC/3TCorTDF/FTC, had no effect on 3^rd^ agent duration. Individualizing treatment can avoid some toxicity discontinuations, e.g. efavirenz and CNS side effects. Use of rilpivirine in naïve patients is associated with long treatment duration if use in patients with high viral load or advanced disease is avoided. The same applies to treatment-experienced patients who switch to rilpivirine with undetectable viral load. In treatment-experienced patients darunavir was the mostly used drug and, besides rilpivirine, also showed the lowest risk for treatment discontinuation.
